# Identification of gut microbes-related molecular subtypes and their biomarkers in colorectal cancer

**DOI:** 10.18632/aging.205480

**Published:** 2024-01-29

**Authors:** Xuliang Liu, Guolin Zhang, Shiyao Li, Yuechuan Liu, Kexin Ma, Liming Wang

**Affiliations:** 1 Department of General Surgery, Division of Hepatobiliary and Pancreatic Surgery, The Second Affiliated Hospital of Dalian Medical University, Dalian, Liaoning, China; 2 Department of Cardiovascular Medicine, The Second Affiliated Hospital of Dalian Medical University, Dalian, Liaoning, China; 3 Department of Respiratory Medicine, The Second Affiliated Hospital of Dalian Medical University, Dalian, Liaoning, China; 4 Engineering Research Center for New Materials and Precision Treatment Technology of Malignant Tumors Therapy, The Second Affiliated Hospital, Dalian Medical University, Dalian, Liaoning, China; 5 Engineering Technology Research Center for Translational Medicine, The Second Affiliated Hospital, Dalian Medical University, Dalian, Liaoning, China

**Keywords:** colorectal cancer, gut microbes, immune infiltration, IL7, BCL10

## Abstract

The role of gut microbes (GM) and their metabolites in colorectal cancer (CRC) development has attracted increasing attention. Several studies have identified specific microorganisms that are closely associated with CRC occurrence and progression, as well as key genes associated with gut microorganisms. However, the extent to which gut microbes-related genes can serve as biomarkers for CRC progression or prognosis is still poorly understood. This study used a bioinformatics-based approach to synthetically analyze the large amount of available data stored in The Cancer Genome Atlas (TCGA) and Gene Expression Omnibus (GEO) databases. Through this analysis, this study identified two distinct CRC molecular subtypes associated with GM, as well as CRC markers related to GM. In addition, these new subtypes exhibit significantly different survival outcomes and are characterized by distinct immune landscapes and biological functions. Gut microbes-related biomarkers (GMRBs), IL7 and BCL10, were identified and found to have independent prognostic value and predictability for immunotherapeutic response in CRC patients. In addition, a systematic collection and review of prior research literature on GM and CRC provided additional evidence to support these findings. In conclusion, this paper provides new insights into the underlying pathological mechanisms by which GM promotes the development of CRC and suggests potentially viable solutions for individualized prevention, screening, and treatment of CRC.

## INTRODUCTION

Colorectal cancer (CRC) ranks as the third most prevalent cancer worldwide and represents a significant global health challenge, being the second leading cause of cancer-related mortality. The incidence and mortality rates of CRC are anticipated to rise substantially, with projections estimating 3.2 million new cases and 1.6 million deaths by 2040, particularly in countries with a high Human Development Index [1–3]. As with many diseases, CRC is the result of the accumulation of multifactorial perturbations that include genetic, epigenetic, and environmental aspects [4]. In particular, environmental factors such as consumption of carcinogenic foods, lack of physical activity, and cigarette smoking are known to assume a pivotal role in the intricate web of CRC progression [5].

Among the environmental factors, there has been increasing recognition of the role of the collective microbial community in the tumor environment. Our colon harbors about 30 trillion bacteria, which constantly interact with our intestinal epithelium. Alterations in the quantity of these microbes can disrupt gut physiology and lead to diseases [6]. Enrichment of several gut microbes (GM), such as *Fusobacterium nucleatum*, *Peptostreptococcus anaerobius*, and *enterotoxigenic Bacteroides fragilis*, has been found to enhance the development of CRC by promoting inflammation [7], DNA damage [8], tumor growth [9], and immune evasion [9]. Conversely, certain bacteria, primarily probiotics including *Bifidobacterium adolescentis* (ATCC 15703) [10] and *Faecalibacterium prausnitzii* (DSM 17677) [11, 12], are observed to be diminished in individuals with CRC and are believed to confer a safeguarding influence against CRC. In addition, there is growing evidence that dysbiosis of gut microbial ecology can influence the development of CRC by affecting the functional state of host cells. Interacting with Toll-like receptors, *Bifidobacterium* leads to the release of interleukin 10 (IL10) and expression of forkhead box P3 (FOXP3) regulatory T cells (Tregs) [13]. Meanwhile, *Lactobacillus rhamnosus GG* suppresses Th17 cell expression and cytokine secretion of interleukin 23(IL23) and interleukin 17(IL17). It also promotes a shift in macrophage phenotype from pro-inflammatory M1 to immunosuppressive M2 by inhibiting nuclear factor kappa B (NF-κB) subunit 1 and signal transducer and activator of transcription 3(STAT3) signaling [14]. Additionally, *Lactobacillus reuteri* activates aryl hydrocarbon receptor (AHR) and influences the levels of interleukin 6(IL6), interleukin 12(IL12), tumor necrosis factor (TNF) and interleukin 7(IL7), as well as macrophage and T-lymphocyte activation status [15]. A recent investigation has revealed that *Bifidobacteria*, known for their protective role against CRC, possess the capability to modulate dendritic cell (DC) maturation as well as tumor-specific T-cell responses. This intriguing finding suggests that *Bifidobacteria* may exert an impactful influence on the therapeutic efficacy of anti-CRC immunization and anti-programmed death ligand 1(PDL1) therapy [16].

Due to the intricate interplay between CRC and gut microbiome, existing studies have predominantly focused on exploring the use of particular microbiota and their metabolites as predictive markers of clinical response or cancer progression through microbiome analysis [17, 18]. However, the contribution of gut microbes-related genes (GMRGs) to the pathogenesis and prognosis of CRC remains unknown, and their potential as immunotherapy targets is yet to be fully understood. In our analysis, we conducted a comprehensive literature review of correlations between gut microbiota and host genes using the PubMed database, resulting in the identification of 164 GMRGs. Based on these genes, we developed a new subtype of CRC, revealing significant differences between various subtypes in terms of survival prognosis, functional enrichment, immune infiltration, and immunotherapy efficacy. Furthermore, we employed univariate and multivariate Cox regression analysis for identifying hub genes highly associated with CRC patient prognosis among GMRGs. By analyzing the expressed core genes in healthy humans, colorectal inflammation patients, and CRC patients, we evaluated their effectiveness as predictive indicators for the occurrence, development, and outcome of CRC.

The core objective of this study is to identify GM-related molecular subtypes and biomarkers to understand the complexity of CRC in terms of molecular biology, immunology, drug sensitivity, survival prognosis, and disease dynamics, aiming to promote more precise treatment planning to improve the survival rate and quality of life of patients.

## RESULTS

### Identification of GM molecular subtypes in CRC

**Figure 1:**
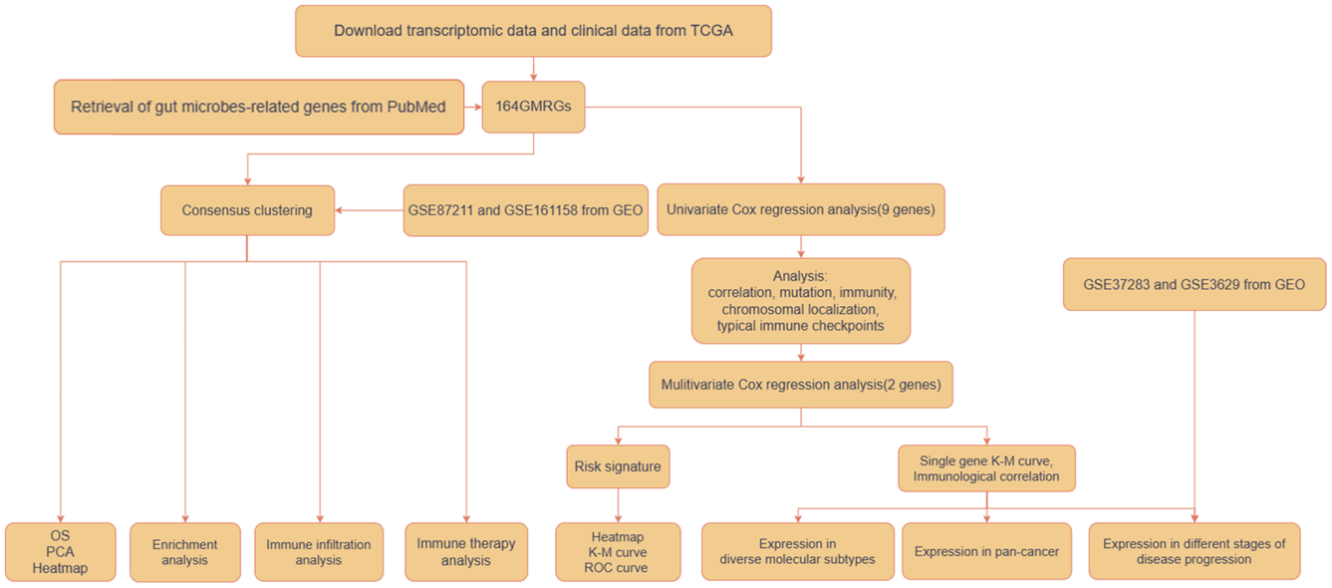
Workflow of the analytic process.

**Figure 2:**
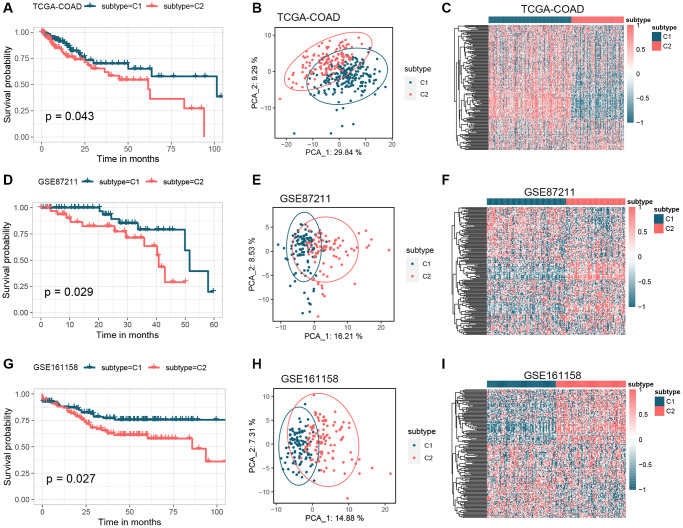
**Identification of GM molecular subtypes in CRC.** (**A**–**C**) K-M analysis, PCA analysis, and heatmap between subtype C1 and subtype C2 in the TCGA-COAD cohort. (**D**–**F**) K-M analysis, PCA analysis, and heatmap between subtype C1 and subtype C2 in the GSE87211 cohort. (**G**–**I**) K-M analysis, PCA analysis, and heatmap between subtype C1 and subtype C2 in the GSE161158 cohort. Abbreviations: GM: gut microbes; CRC: colorectal cancer; K-M: Kaplan-Meier; PCA: principal component analysis.

Figure 1 shows how the study works. A total of 164 GMRGs were searched and screened in the PubMed database, and their expression levels were compared between 332 cancer and 41 normal samples from the TCGA-COAD dataset (Supplementary Figure 1). A noticeable discrepancy in transcript levels was observed between the two subgroups, as shown in the heatmap. The principal component analysis (PCA) plot further confirmed the existence of differential expression of GMRGs. Using a consensus clustering algorithm, the 332 CRC patients were classified into two subtypes with optimal cluster stability (K = 2) (Supplementary Figure 2). Among them, 203 patients were assigned to the C1 cluster and 129 to the C2 cluster. The Kaplan-Meier (K-M) analysis revealed that overall survival (OS) was significantly longer in subtype C1 compared to subtype C2 (*P* = 0.043, Figure 2A). The transcriptomes of the two subtypes were significantly different, as shown by PCA (Figure 2B) and heatmap (Figure 2C). To validate the results, the GSE87211 dataset was used as a validation set, which showed similar results (Figure 2D–2F). Similarly, the GSE161158 dataset was used as a test set, and the consensus clustering results confirmed that the C1 subtype had a better prognosis than the C2 subtype (*P* = 0.027, Figure 2G). The PCA (Figure 2H) and heatmap (Figure 2I) analyses also demonstrated significant differences between the two subtypes.

### Functional annotation of GM molecular subtypes

**Figure 3:**
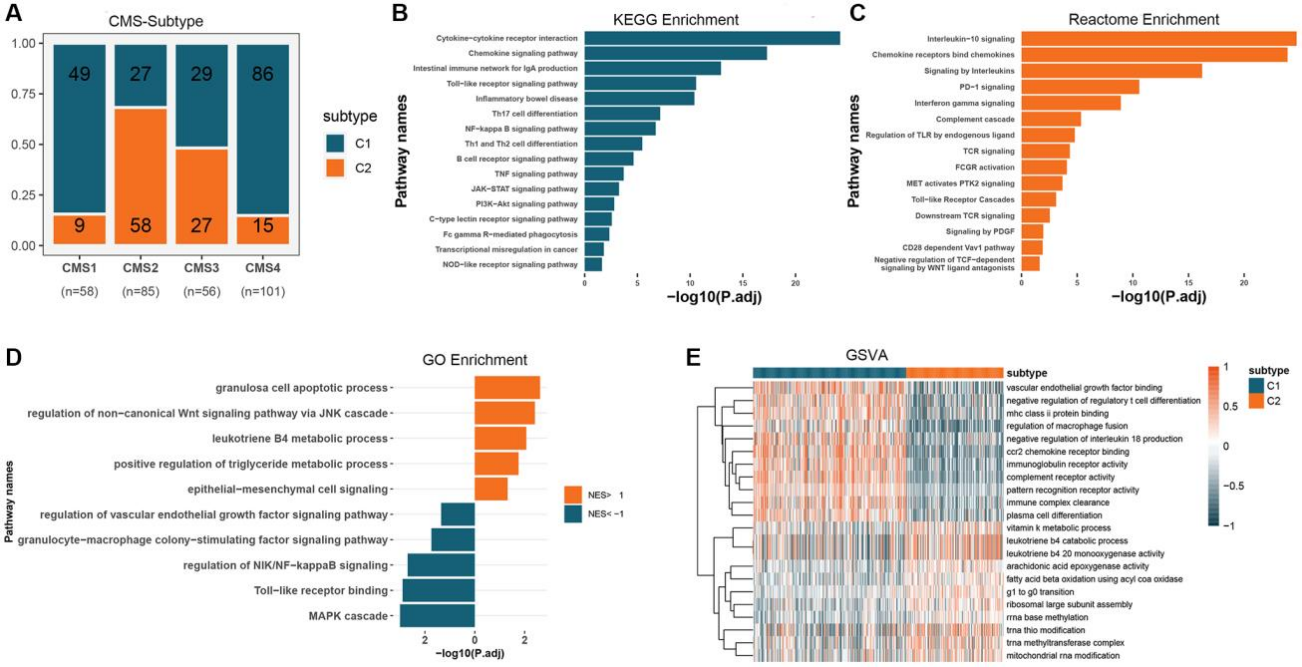
**Functional expression patterns of GM molecular subtypes in CRC.** (**A**) Distribution of GM molecular subtypes in CRC consensus subtypes. (**B**) KEGG enrichment analysis of DEGs in GM molecular subtypes. (**C**) Reactome enrichment analysis of DEGs in GM molecular subtypes. (**D**) GSEA analysis of GM molecular subtypes using the GO database. (**E**) GSVA analysis of GM molecular subtypes using the GO database. Abbreviations: GM: gut microbes; CRC: colorectal cancer; KEGG: Kyoto Encyclopedia of Genes and Genomes; DEGs: differentially expressed genes; GSEA: gene set enrichment analysis; GO: Gene Ontology; GSVA: gene set variation analysis.

To understand the functional patterns of two colon cancer subtypes based on GMRGs expression, we analyzed their distribution across the consensus molecular subtypes (CMS) of CRC (Figure 3A). We observed that C1 subtypes were more prevalent in CMS1 and CMS4 than C2 subtypes, while C2 subtypes were more prevalent in CMS2. To gain insight into the potential associations between these two classifications and aid in clinical translation, we conducted functional enrichment. We performed a differential analysis of the two subtypes to obtain differentially expressed genes (DEGs) in the different types (Supplementary Figure 3). Kyoto Encyclopedia of Genes and Genomes (KEGG) and Reactome enrichment analysis revealed that GMRGs were primarily enriched in the developmental maturation of the immune system and intra- and extracellular signaling, among others (Figure 3B, 3C). Using the Gene Ontology (GO) database for gene set enrichment analysis (GSEA) analysis (Figure 3D), we found that enriched pathways in the C1 subtype were mainly associated with the regulation of inflammatory immune response, cellular responses to various stimuli, and angiogenesis regulation. Meanwhile, enriched pathways in the C2 subtype were mainly associated with Wnt signaling, epithelial-mesenchymal transition, apoptosis, and metabolic regulation. We further performed gene set variation analysis (GSVA) analysis using the GO database (Figure 3E), which showed that enriched pathways in the C1 subtype were mainly associated with immune regulation, including macrophage fusion, T cell and plasma cell differentiation, and binding of vascular endothelial growth factor and its regulation. The enriched pathways in the C2 subtype were mainly associated with ribosome assembly, biological enzyme activity, and cell cycle regulation.

### Evaluation of immune features and immunotherapy for GM molecular subtypes

**Figure 4:**
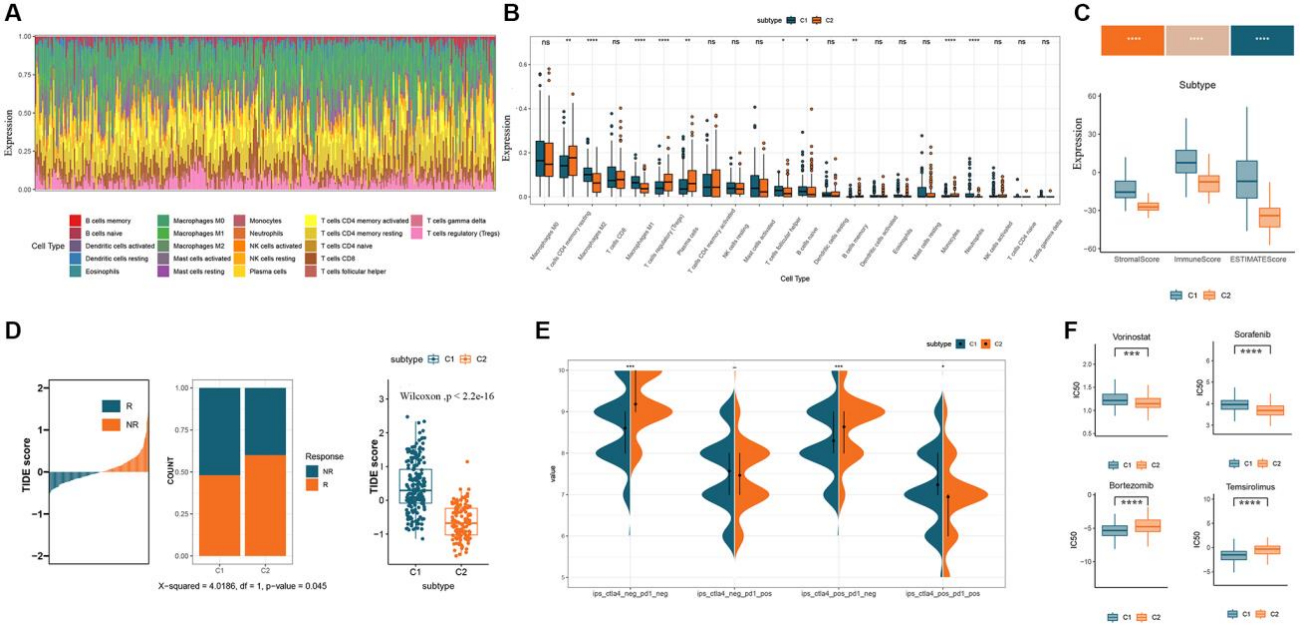
**Immunological characterization and evaluation of immunotherapy for GM molecular subtypes.** (**A**) Relative content distribution of 22 TICs in COAD samples. (**B**) Box plots showing the difference in abundance of 22 TICs between the two subtypes. (**C**) Comparison of stromal, immune, and ESTIMATE scores in the two subtypes. (**D**) Estimation of immunotherapy effect in the two subtypes using the TIDE algorithm. (**E**) Immune checkpoint expression in two GM subtypes. (**F**) Box plots of the estimated IC50 of chemotherapy drugs between the two GM subtypes. Abbreviations: GM: gut microbes; TICs: tumor-infiltrating immune cells; TIDE: Tumor Immune Dysfunction and Exclusion; IC50: semi-inhibitory concentration.

To further characterize tumor microenvironment (TME) in GM subtypes, we conducted CIBERSORT analysis to evaluate the distribution of 22 tumor-infiltrating immune cells (TICs) in the COAD cohort (Figure 4A). Significant disparities in the abundance of distinct immune cell populations were observed between the C1 and C2 subtypes. The C1 subtype showed higher levels of M1 macrophages, T cells follicular helper, and B cells native compared to the C2 subtype. In contrast, regulatory T cells, plasma cells, and monocytes were more abundant in the C2 subtype compared to the C1 subtype (Figure 4B). To further evaluate TME, the ESTIMATE algorithm was employed to calculate the ESTIMATE score, immune score, and stromal score. The findings revealed that the C1 subtype exhibited a significantly elevated ESTIMATE score (*P* < 0.001), immune score (*P* < 0.001), and stromal score (*P* < 0.001) in comparison to the C2 subtype (Figure 4C). Subsequently, the Tumor Immune Dysfunction and Exclusion (TIDE) algorithm was utilized to predict the response to immunotherapy in CRC patients with varying levels of immune infiltration and GM subtypes. By stratifying responders and non-responders based on the median TIDE score, a higher proportion of responders was observed in the C2 subtype characterized by low immune infiltration and low TIDE scores (Figure 4D). This suggests that the C2 subtype, characterized by lower immune infiltration, may exhibit a more favorable response to immunotherapy. Furthermore, we examined the expression of immune checkpoint markers as potential indicators of immunosuppression. The analysis revealed a higher proportion of samples in the C1 subtype with high expression of programmed cell death 1 (PD1) and cytotoxic T-lymphocyte associated protein 4 (CTLA4), while the C2 subtype had a higher proportion of samples with low expression of immune checkpoint markers (Figure 4E). Lastly, we performed drug sensitivity predictions to identify potential therapeutic options for each subtype. The analysis showed that the C2 subtype was more sensitive to Vorinostat and Sorafenib, while the C1 subtype exhibited higher sensitivity to Bortezomib and Temsirolimus (Figure 4F).

### Identification and analysis of prognosis-related GMRGs

**Figure 5:**
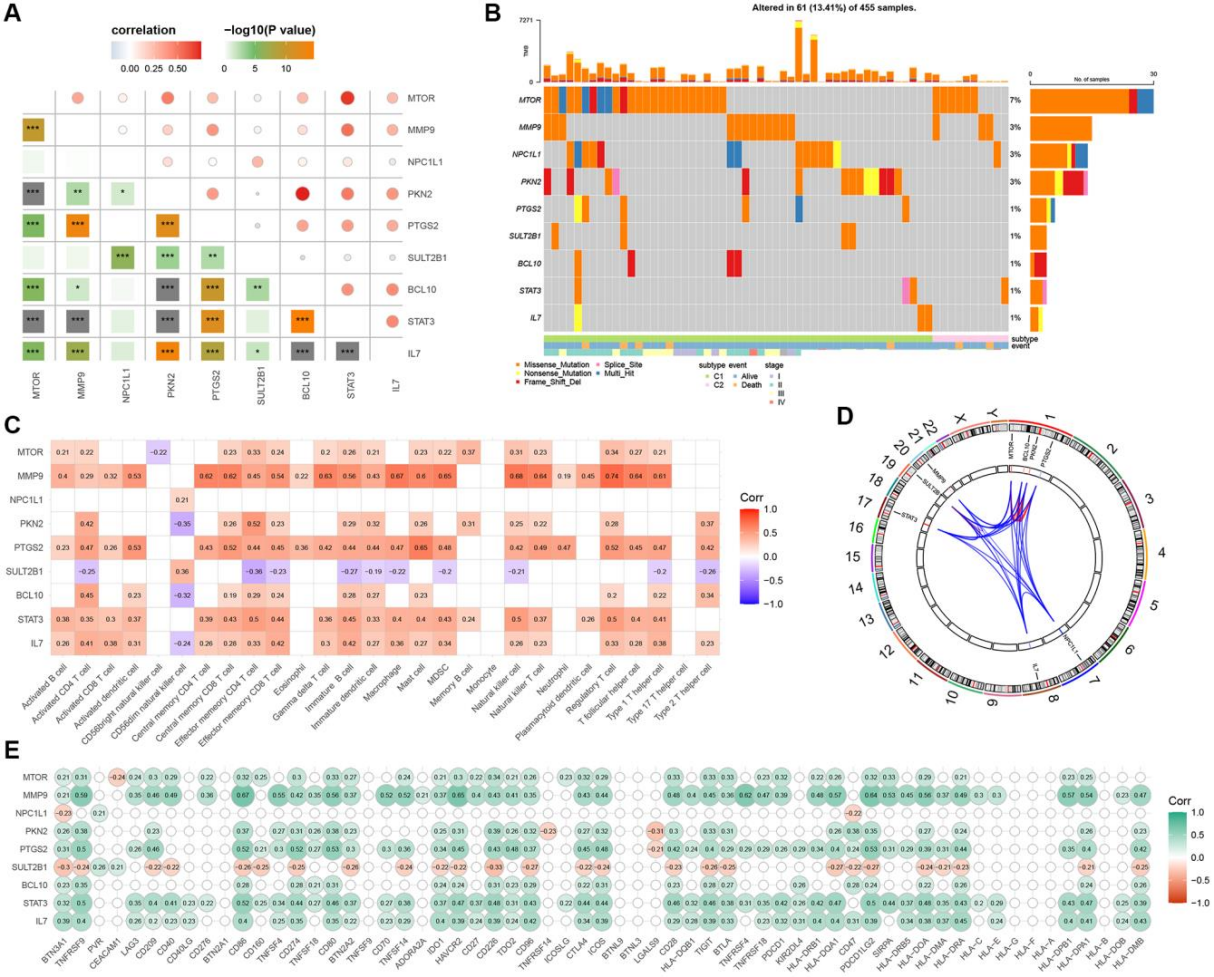
**Discovery and investigation of GMRGs linked to prognostic outcomes.** (**A**) Correlation analysis of 9 prognostic genes obtained by univariate Cox regression analysis. (**B**) Mutation landscape of 9 prognostic genes. (**C**) Correlation analysis of 9 prognostic genes with immune cells. (**D**) Display of 9 prognostic genes locations on the chromosome. (**E**) Correlation analysis of 9 prognostic genes with immune checkpoints. Abbreviation: GMRGs: gut microbes-related genes.

We conducted univariate Cox regression analysis on 164 GMRGs and identified 9 prognostic genes in the TCGA-COAD cohort (*P* < 0.05) (Supplementary Table 1). Subsequently, we examined the correlation between these 9 genes (Figure 5A) to assess their association. We also analyzed the somatic mutation frequencies of these 9 GMRGs and found relatively high mutation rates in the TCGA-COAD samples (Figure 5B). Among the samples, 61 (13.41%) had mutations in the GMRGs, with MTOR showing the highest mutation frequency (7%), followed by MMP9, NPC1L1, PKN2, PTGS2, SULT2B1, BCL10, STAT3, and IL7. Furthermore, we explored the relationship between these 9 prognostic genes and immune cells to investigate the interplay between gene expression and immune functions (Figure 5C). We also determined the chromosomal locations of these genes (Figure 5D). To examine whether changes in gene expression directly impact checkpoint activity or vice versa, we assessed the correlation between the 9 prognostic genes and immune checkpoints (Figure 5E). Interestingly, SULT2B1 exhibited a significant negative correlation with most of the checkpoints, while MMP9 showed a strong positive correlation with most of the immune checkpoints.

### Construction and evaluation of GM risk signature

**Figure 6:**
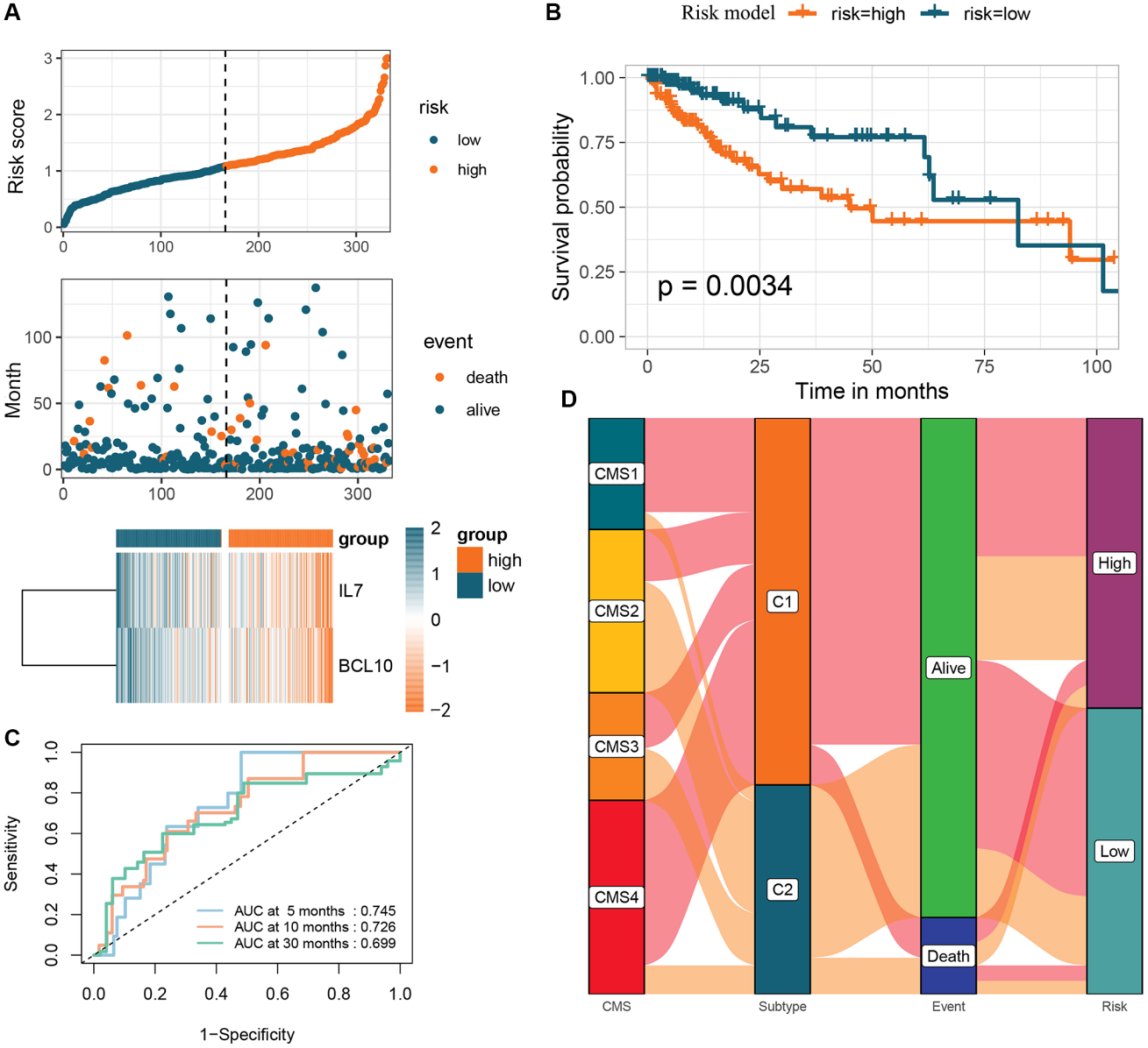
**Creation of a risk profile through stepwise multiple Cox regression analysis based on two GMRGs.** (**A**) Analysis of GMRG risk scores: distribution of risk scores among CRC patients; survival status and duration of CRC patients; heatmap illustrating the expression of 2 genes. (**B**) K-M survival analysis of CRC patients in the high and low-risk groups in the TCGA-COAD cohort. (**C**) ROC calibration curves predicting overall survival at 5, 10, and 30 months in the high- and low-risk groups. (**D**) Sankey diagram illustrating the differential distribution of CRC patients in CRC consensus molecular subtypes, GM subtypes, survival outcomes, and high- and low-risk groups. Abbreviations: GMRGs: gut microbes-related genes; CRC: colorectal cancer; K-M: Kaplan-Meier; ROC: receiver operating characteristic; GM: gut microbes.

The prognostic signature based on 2 GMRGs was established using stepwise multivariate Cox regression analysis (Figure 6A). The prognostic model formula was as follows: risk score = (−0.044870355898046 × expression level of IL7) + (−0.00841862944423366 × expression level of BCL10). Using the median risk score, CRC patients were divided into high-risk (*n* = 166) and low-risk (*n* = 166) groups. The high-risk group showed a lower long-term survival rate compared to the low-risk group (*p* = 0.0034, Figure 6B). The sensitivity and specificity of the prognostic characteristics were assessed using receiver operating characteristic (ROC) curves for 5-, 10-, and 30-month overall survival rates, with area under the curves (AUCs) of 0.745, 0.726, and 0.699, respectively (Figure 6C). Forest plots were used to present the results of the clinical characteristics analysis (Supplementary Figure 4). The relationship between risk models, GM molecular subtypes, CRC consensus molecular subtypes, and the end state was analyzed to gain a deeper understanding of disease mechanisms (Figure 6D).

### GMRBs survival analysis and immune correlation analysis

**Figure 7:**
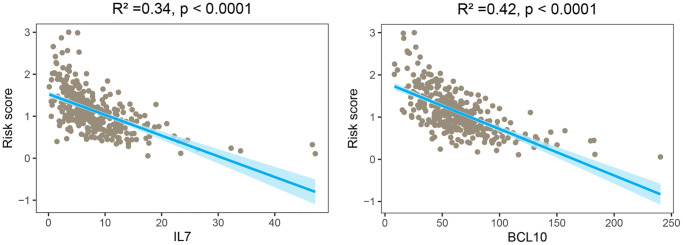
**Correlation analysis between risk scores and expression levels of the two GMRBs.** The scatter plot from the correlation analysis revealed a significant negative correlation between the risk score and the expression levels of the two GMRBs (IL7 and BCL10). Abbreviation: GMRBs gut microbes-related biomarkers.

**Figure 8:**
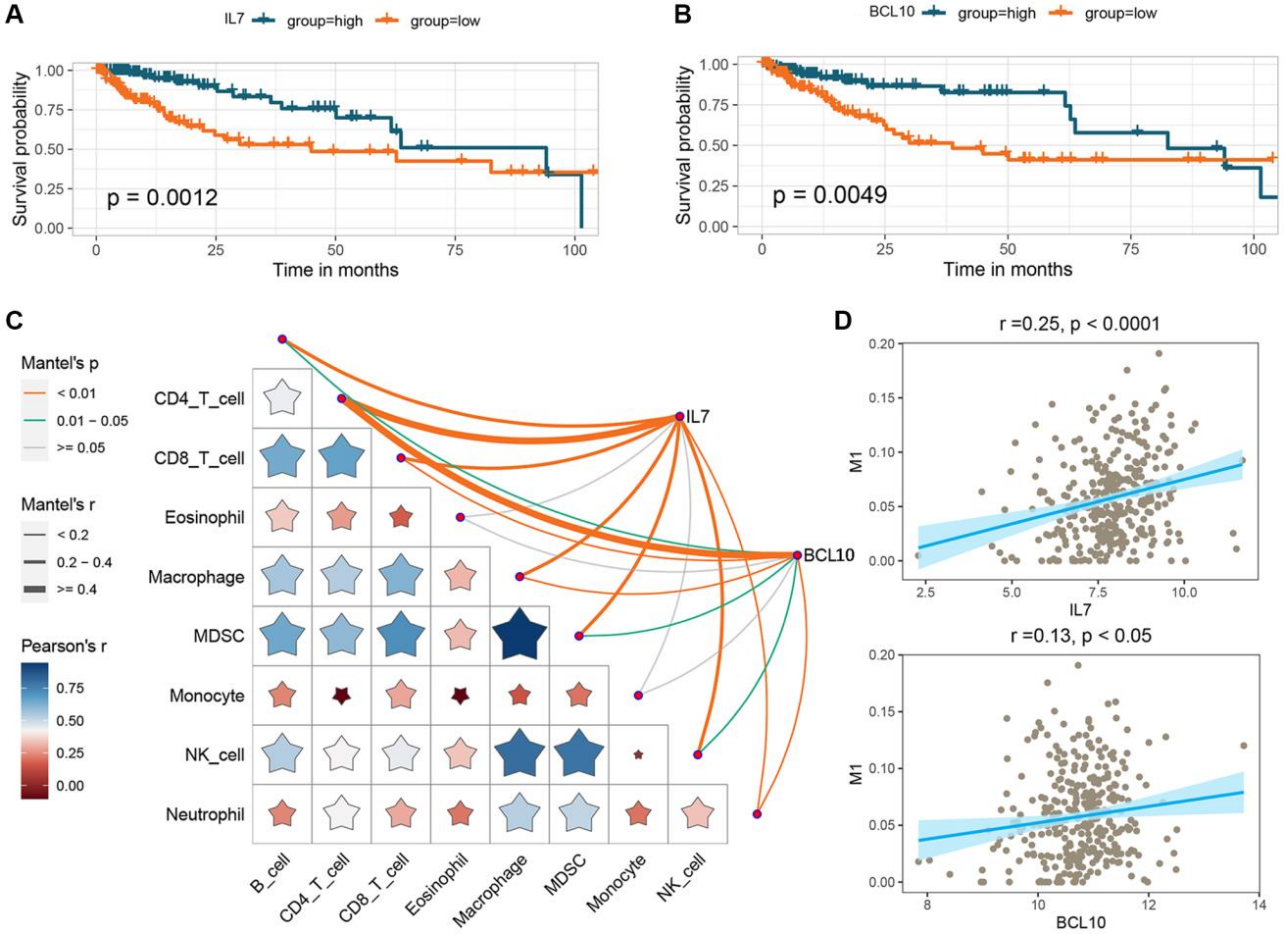
**Analysis of the prognostic impact of GMRBs and their correlation with immune response.** (**A**) K-M analysis of OS in CRC patients based on IL7 expression. (**B**) K-M analysis of OS in CRC patients based on BCL10 expression. (**C**) Correlation analysis of GMRBs with immune cell infiltration. (**D**) Correlation analysis of GMRBs with M1 macrophages. Abbreviations: GMRBs: gut microbes-related biomarkers; K-M: Kaplan-Meier; OS: overall survival; CRC: colorectal cancer.

To investigate the potential prognostic significance of the two GMRBs identified by multivariate Cox regression analysis, we conducted a correlation analysis between risk scores and the expression levels of GMRBs. Notably, a significant negative correlation was observed between IL7 and BCL10 and risk scores (Figure 7). Based on the median gene expression as a threshold, patients were categorized into high and low-expression groups. Subsequent survival analysis was performed for each group, and K-M survival curves were generated to visually represent these findings (Figure 8A, 8B). The prognostic characteristics of both IL7 and BCL10 consistently demonstrated a better prognosis in the high-expression group. To further validate the prognostic characteristics, we performed an analysis in an independent GEO cohort, confirming the favorable prognosis associated with high expression of both IL7 and BCL10 (Supplementary Figure 5). Moreover, to elucidate the underlying biological mechanisms, we investigated their correlation with immune cell populations. Remarkably, both IL7 and BCL10 exhibited a strong association with CD4 T cells (Figure 8C). Moreover, both GMRBs displayed a positive correlation with M1 cell expression (Figure 8D), indicating the potential involvement of GMRBs in its activation or recruitment.

### Expression analysis of GMRBs across diverse molecular subtypes, cancer types, and disease stages

**Figure 9:**
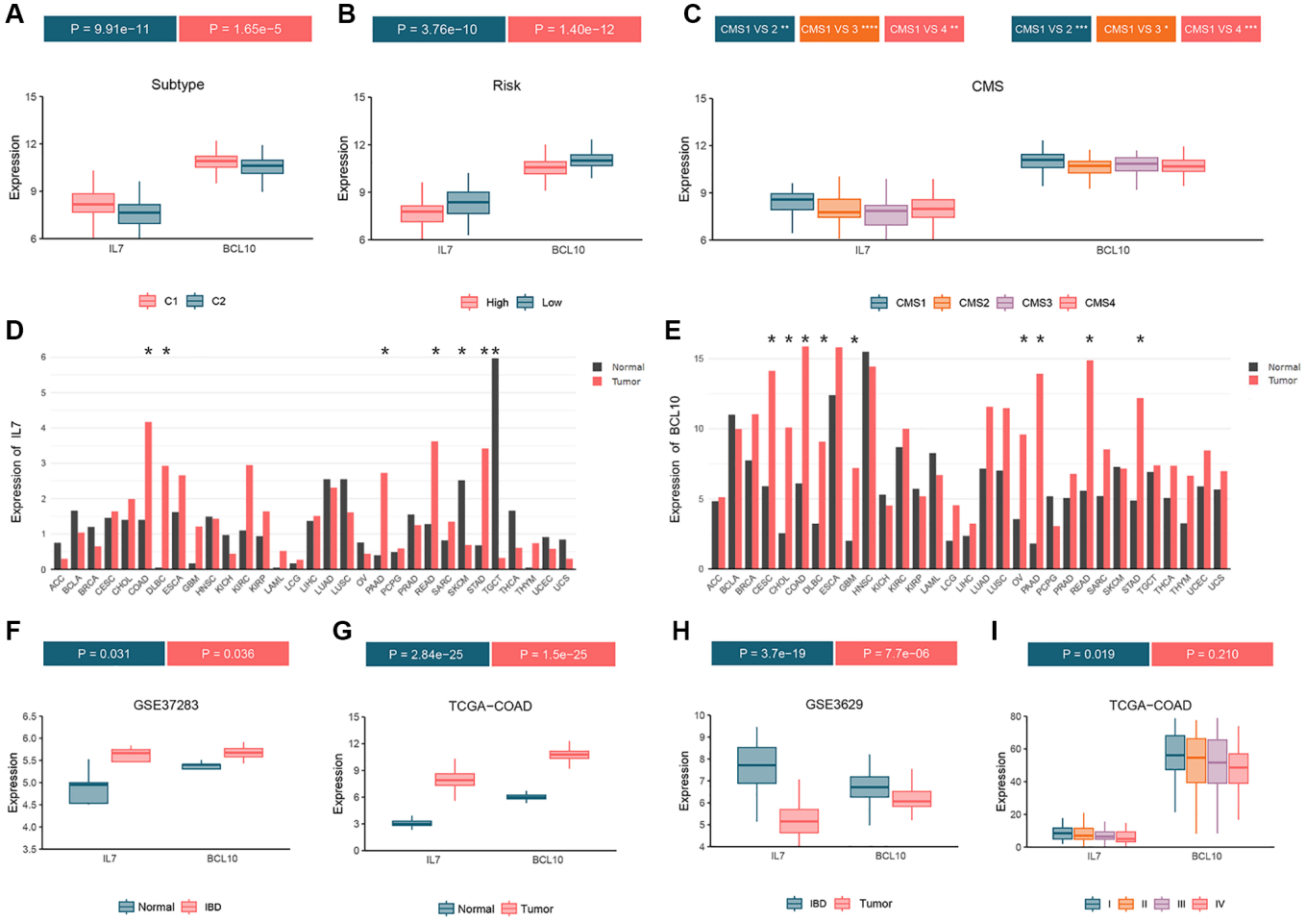
**Analyze the expression status of GMRBs in different molecular subtypes, cancer types and disease stages.** (**A**) GMRBs expression in molecular subtypes obtained by consensus clustering. (**B**) GMRBs expression in high-risk and low-risk groups. (**C**) GMRBs expression in CRC consensus molecular subtypes. (**D**, **E**) Expression of IL7 and BCL10 in various cancer types. (**F**) Differential expression of GMRBs between healthy individuals and patients with IBD. (**G**) Differential expression of GMRBs between healthy individuals and patients with CRC. (**H**) Differential expression of GMRBs between patients with IBD and patients with CRC. (**I**) Differential expression of GMRBs in CRC patients at different disease stages. Abbreviations: GMRBs: gut microbes-related biomarkers; CRC: colorectal cancer; IBD: inflammatory bowel disease.

Differential expression analysis of the two GMRBs was performed across various molecular subtype systems, including consensus clustering (Figure 9A), high- and low-risk clustering (Figure 9B), and CRC consensus subtypes (Figure 9C). The analysis revealed higher expression levels of both IL7 and BCL10 in the C1 subtype compared to the C2 subtype. Furthermore, in the high-risk group, their expression was lower than in the low-risk group. Notably, the CMS1 subtype, characterized by a strong immune profile in CRC consensus subtypes, exhibited the highest expression of GMRBs. Pan-cancer analysis revealed that GMRBs showed differential expression in different cancers (Figure 9D, 9E). Interestingly, in the COAD, DLBC, PAAD, READ, and STAD datasets, both IL7 and BCL10 were expressed higher in the cancer group than in the normal group. Additionally, differential expression data of the GMRBs were obtained for healthy individuals, patients with inflammatory bowel disease (IBD), and CRC patients using the GSE37283, TCGA-COAD, and GSE3629 datasets. Both IL7 and BCL10 exhibited elevated expression levels in both IBD and CRC patients compared to healthy individuals (Figure 9F, 9G). When comparing the IBD and CRC groups, both genes showed higher expression levels in the CRC group (Figure 9H). Finally, in the TCGA-COAD dataset, the expression levels of the GMRBs were analyzed in patients at different stages of CRC. The analysis showed that the expression of GMRBs showed a decreasing trend at different stages of the cancer (Figure 9I).

## DISCUSSION

This study provides a comprehensive analysis of the role of GMRGs in CRC, including their subtyping, prognostic significance, immune-related characteristics, and potential as predictive markers. Initially, we examined the differential expression of 164 GMRGs in the TCGA-COAD dataset, comparing cancer and normal groups. Using a consensus clustering algorithm, we identified two distinct GM clusters, namely subtype C1 and subtype C2, in the TCGA-COAD cohort. These clusters were further validated using the GEO cohort. Through comprehensive functional enrichment and immune analysis, we embarked on a profound exploration of the intricate biological functionalities and immunological characteristics exhibited by each cancer subtype. By conducting univariate Cox regression analysis, we found that nine genes (MTOR, MMP9, NPC1L1, PKN2, PTGS2, SULT2B1, BCL10, STAT3, IL7) exhibited significant associations with OS within CRC patients. We also examined characteristics such as mutations and immunity in these genes. Further analysis using stepwise multifactorial Cox regression identified two selected OS-related GMRBs, namely IL7 and BCL10. Prognostic models were developed based on these GMRBs, allowing for clinical survival predictions. Additionally, we investigated the association of IL7 and BCL10 with prognosis and immunity. We explored their expression levels across different staging systems, cancer types, disease stages, and CRC stages. In summary, our study revealed novel insights into the GM molecular subtypes of CRC and identified IL7 and BCL10 as predictive GM markers for CRC.

Molecular subtyping of tumors has garnered considerable attention in cancer research due to its relevance in prognostic prediction and understanding the composition of the TME. However, there remains a limited number of comprehensive studies investigating the role of GMRGs in molecular subtypes of CRC. Previous research by Zheng et al. focused on stem cell-related molecular subtypes associated with CRC prognosis [19], while Dai et al. established a senescence-related subtype using 91 senescence-related genes [20]. Additionally, Yuan et al. employed a non-negative matrix factorization clustering algorithm to identify energy metabolism-related CRC subtypes [21]. In this study, we aimed to identify distinct GM molecular subtypes (C1 and C2) based on their expression profiles in CRC. Our findings revealed a strong association between the C1 subtype and CMS1 and CMS4 subtypes, representing immune activation and stromal invasion, respectively, as defined by Justin et al. [22]. Furthermore, enrichment analysis demonstrated that C1 subtypes have significant regulatory effects on immune responses and immune system processes, particularly in Toll-like receptor (TLR) signaling, mitogen-activated protein kinase kinase kinase 14 (MAP3K14)/NF-κB signaling, and vascular endothelial growth factor signaling pathways. TLR signaling is critical for innate and adaptive immunity, as it plays a crucial role in the development and maturation of the human immune system [23]. This receptor detects pathogen-associated molecular patterns, activating the immune system and providing opportunities for the use of tailored adjuvants with specific immune effects in targeted cancer therapy [24].

Regarding TME patterns, the C1 subtype exhibited a substantial enrichment of M1 macrophages known for their pro-inflammatory and anti-tumor properties [25]. These M1 macrophages possess enhanced phagocytic activity and secrete pro-inflammatory cytokines and chemokines that facilitate the recruitment and activation of cytotoxic T cells and natural killer cells, effectively targeting and eliminating malignant cells [26]. Additionally, M1 macrophages promote tumor antigen presentation and support the generation of a robust anti-tumor immune response [27]. Stimulators of the classical pathway, such as bacterial components, interferon-gamma, lipopolysaccharide, and TLRs, promote the polarization of M1 macrophages. These polarized M1 macrophages induce oncogenic effects by releasing pro-inflammatory cytokines (e.g., interleukin 1 and IL12) and cytotoxic agents (e.g., reactive oxygen species and TNF) [28, 29]. In addition, the C2 subtype exhibited a significant enrichment of Tregs, which play a complex role in the progression and immune evasion of CRC [30]. Tregs, characterized by FOXP3 expression, are recruited to CRC sites via chemokine signaling and exert immunosuppressive functions by suppressing effector T cell responses and hindering anti-tumor immune surveillance [31]. Mechanistically, Tregs exert their suppressive effects through the secretion of immunosuppressive cytokines such as IL10 and transforming growth factor-β (TGF-β), as well as direct cell-to-cell contact involving immune checkpoint molecules such as CTLA4 [32, 33]. The abundance of Tregs within CRC tumors is associated with a poor prognosis and reduced patient survival [34]. Notably, our study found that the outcome of immune infiltration in both GM molecular subtypes was consistent with prognosis, with patients characterized by TME activation in the C1 subtype exhibiting a longer survival time than those with TME suppression in the C2 subtype.

Based on GM molecular subtypes, we identified 2 core GMRGs (IL7 and BCL10). IL7 is an important cytokine that can be regulated by gut microorganisms and is involved in human immune response and lymphocyte development [35–37]. Intestinal symbionts with preventive or inhibitory effects on colorectal cancer, *Bifidobacterium* [38] and *Lactobacillus reuteri* [39], have been disclosed to have significant regulatory effects on host IL7 expression [35, 40]. Among them, a specific subspecies of *Lactobacillus reuteri*, *clade IV strains*, was able to specifically activate human myeloid cells and significantly increase their IL7 expression [40]. Current research substantiates the vital role of IL7 throughout all phases of T-cell development [41, 42] and highlights its importance in the preservation of quiescent T cells and the establishment and sustenance of memory T cell populations [43, 44]. The restoration of T cell homeostasis through the activation of the suppressor of cytokine signaling (SOCS)/signal transducer and activator of transcription 5 (STAT5) pathway [45] has been demonstrated for IL7, alongside its role in guiding T cell migration, homing and activation through the induction of chemokine and integrin expression [46, 47]. Activated T cells then mediate the tumor antigen recognition process of CRC immunotherapy, a key process that greatly determines the efficacy and availability of antigen-targeted immunotherapy for cancer patients [48, 49]. In particular, it should be emphasized that owing to extensive and potent biological effects of IL7 on T-cell survival, development, proliferation, and memory maintenance, several studies have employed it as a molecular adjunct to augment the antigenicity elicited by cancer vaccines and sustain long-term memory responses against cancer. Zhao et al. reported persistent and active IL7 production in cancer vaccines modified with the IL7 gene, with undetectable levels in tumor cells [50]. The supernatant from transfected cancer cells was found to enhance T-cell proliferation, with a volume of 10 μl corresponding to 1 ng of recombinant human IL7 protein [46]. Whole tumor cell-based vaccines expressing IL7 demonstrated vigorous preventative and therapeutical outcomes in murine models, inhibiting tumorigenesis and extending survival time. These vaccines also increased T-lymphocytes of the CD4 and CD8 subsets infiltration within tumor fields [50, 51]. In a Phase-II clinical trial, subcutaneous injection of IL7 in oncology patients under treatment with the FDA-approved tumoral vaccine sipuleucel-T resulted in the augmentation of CD4 and CD8 T lymphocytes [52, 53]. Enhanced levels of intracellular cytokines, such as interleukin 2(IL2), TNF-α, interferon-gamma (IFN-γ), and IL6, were observed within the memory subpopulation, indicating that IL7 may stimulate a memory immune response targeting cancer. Furthermore, Jeong et al. investigated the delivery of a fusion protein containing human macrophage migration inhibitory factor (MIF) and IL7 through *Mycobacterium smegmatis*, utilized as a bacterial-based cancer vaccine [54]. This vaccine elicited anti-tumor immune responses through the recruitment of potent T cells and reduced the infiltration of myeloid-derived suppressor cells (MDSCs) within the TME. Notably, combining this vaccine with PDL1 immunotherapy led to heightened antitumor effects. Furthermore, in our study, we consistently observed elevated IL7 expression in the immune inflammation-activated phenotype C1. This higher expression was significantly associated with CD4 T cell infiltration in TME and correlated with a favorable prognosis in the high-expression group. When comparing the normal, IBD, and cancer groups, we found that IL7 levels were higher in the cancer group compared to the control group but lower than in the IBD group. In conclusion, the above findings not only reveal that IL7 regulated by GM has the potential to serve as a cancer vaccine adjuvant and a novel immune checkpoint in CRC, but also emphasize the great feasibility of in-depth study of certain specific gut-microbial subspecies including *Lactobacillus reuteri clade IV strains* for the realization of microecological therapies for CRC.

BCL10, a critical member of the caspase activation and recruitment domain (CARD) family, plays a pivotal role in mediating inflammatory responses [55]. Through interactions with CARD11 and mucosa-associated lymphoid tissue lymphoma translocation protein 1 (MALT1), it forms the CARD11-BCL10-MALT1 (CBM) complex [56]. Activation of the CBM complex downstream of B-cell receptor (BCR) or T-cell receptor (TCR) signaling in B and T cells initiates the NF-κB and mitogen-activated protein kinase (MAPK) pathways, leading to a robust phenotypic response [57, 58]. In particular, hyperactivation of the NF-κB pathway permits tumor-selective reprogramming of the chemokine microenvironment, and this reprogramming greatly enhances the recruitment of effector CD8(+) T cells, which in turn leads to the generation of potent anti-CRC effects [59, 60]. Notably, butyrate produced by intestinal *Roseburia intestinalis* can bind directly to T cell surface receptors, which would likely induce CD8 T cell cell cell activation through CBM complex activation of NF-κB signaling [61]. In addition, Aberrant CBM function resulting from BCL10 mutations is a key factor in a range of diseases, including B-cell lymphoma, autoimmune diseases, and IBD [62–64]. Xia et al. reported frequent mutations in BCL10 signaling mediators, which chronically activate the CBM signaling amplification complex, thereby contributing to an unfavorable outcome in activated B-cell-like diffuse large B-cell lymphoma (ABC-DLBCL) [62]. Similarly, Zhou et al. demonstrated a significant positive correlation between higher expression levels of BCL10 and favorable survival outcomes in colon cancer patients [65]. Furthermore, Borthakur et al. found that carrageenan, a high molecular weight sulfated polygalactan, significantly increases BCL10 secretion in patients with IBD [66]. Collectively, these studies underscore BCL10 as a valuable prognostic indicator in CRC patients. The enriched NF-κB and MAPK pathways, along with its association with T and B cells, suggest that BCL10 may exert its effects by inducing an inflammatory response enhancing killing and inhibiting tumor cell proliferation, further emphasizing its potential significance.

Although our study has yielded encouraging results, there are several specific limitations that should be addressed. Firstly, while we utilized open data sources including TCGA and GEO to construct, validate, and test GM subtypes, we acknowledge the necessity for external independent large datasets with comprehensive coverage to facilitate further in-depth investigations. Secondly, the lack of complete and reliable experimental evidence poses challenges in validating the actual impact of hub genes in clinical immunotherapy and comprehending the influence of microecological modulators on hub gene expression levels. In addition, the analysis of the relationship between GM and host genes in the paper relied primarily on existing literature, and further experiments may be needed to determine the specific mechanisms linking GM, GMRBs, and CRC. Furthermore, it is imperative to acknowledge that the exact mechanism underlying the role of GM in CRC remains unclear, and the fluctuation in the amount and ratio of pathogenic and non-pathogenic GMs in different genera is often implicated in the development and progression of CRC through its involvement in the role of GM. Therefore, further research in this area is warranted. Future studies should endeavor to elucidate the intricate mechanisms underlying the involvement of these two GMRBs in CRC progression and to meticulously evaluate their efficacy in the context of clinical immunotherapy and microecological adjuvant therapy for CRC.

In conclusion, this paper presents novel CRC subtypes and biomarkers associated with GM, thereby providing new potentially feasible options for patient risk stratification and immunotherapy.

## MATERIALS AND METHODS

### Data acquisition

We downloaded RNA sequencing (RNA-seq) information and clinical information of CRC patients from The Cancer Genome Atlas (TCGA; https://portal.gdc.cancer.gov/) database, which was processed to obtain 332 cancer samples as well as 41 normal samples. Then, the GSE87211, GSE161158, GSE37283, and GSE3629 datasets were collected from the Gene Expression Omnibus (GEO; https://www.ncbi.nlm.nih.gov/geo/) project. The GSE87211 and GSE161158 datasets were used as external validation sets, while the GSE37283 and GSE3629 datasets contained the expression profiles of patients with colitis. In addition, somatic mutation data were acquired from TCGA, containing 455 CRC samples.

### Consensus clustering of GMRGs

We employed a range of keyword search strategies in the PubMed database, including “Medical Subject Headings (MeSH)”, “probiotics”, “microorganisms”, “microbiome”, “microbiome”, “bacteria”, and “fungi” in conjunction with “host”, “human”, “intestinal”, “colitis”, “Crohn’s disease”, “ulcerative colitis”, “colorectal”, and “colon cancer” term combinations to retrieve related articles. The reference lists of relevant articles were also reviewed, resulting in 164 GMRGs, as detailed in Supplementary Table 2. Based on the GMRGs expression levels, we applied consensus unsupervised clustering to categorize patients into separate GM molecular subtypes using the “ConsensusClusterPlus” R package [67]. Then, we compared the overall survival (OS) of GM molecular subtypes with the assistance of the R packages “survminer” and “survival”. Principal component analysis (PCA) and heatmap visualization were conducted to show the classification of GM molecular subtypes. Subsequently, it was further validated and tested on external datasets GSE87211 and GSE16115.

### Association analysis of GM molecular subtypes with consensus molecular subtypes of CRC

To classify the CRC patient samples into consensus subtypes, we employed the “Biobase” and “CMScaller” packages in R. We then visualized the proportion of GM molecular subtypes in each consensus subtype using the “tidyverse” and “ggplot2” packages. This approach allowed for a comprehensive exploration of the characteristics of the GM molecular subtypes and their relationship with consensus subtypes.

### Differential expression analysis and functional enrichment of GM molecular subtypes

The limma, DESeq2, and edgeR packages in R were utilized to determine differentially expressed genes (DEGs) within dissimilar GM molecular subtypes. The significant criterion for identifying DEGs was set to |log2 (FoldChange)| > (mean(abs(log2FoldChange)) + 2 × sd(abs(log2FoldChange))) and adjusted *P*-value < 0.05. The “ReactomePA” and “org.Hs.eg.db” R packages were used for the Kyoto Encyclopedia of Genes and Genomes (KEGG) and Reactome enrichment analyses of DEGs.

### Gene set enrichment analysis (GSEA) and gene set variation analysis (GSVA)

The gseGO function of “clusterProfiler” package was deployed to examine the underlying mechanism of diverse GM molecular subtypes, subsequently identifying enriched Gene Ontology (GO) pathways. To discern dissimilar biological functions among GM molecular subtypes, GSVA was executed on “c5.go.bp.v7.5.symbols” using the “GSVA” R package. Visualization of results was carried out via implementation of the “pheatmap” R package.

### Immune cell infiltration analysis

We used CIBERSORT deconvolution algorithm for quantifying abundance of tumor-infiltrating immune cells (TICs) in each CRC sample [68]. Specifically, we downloaded the TICs gene expression signature matrix on the CIBERSORT website platform (https://cibersortx.stanford.edu/) and employed it in our analysis. This allowed us to accurately determine the composition and quantity of TICs present in each sample and provided valuable insights into the immune microenvironment of CRC. Then, we used the R package “ESTIMATE” to assess the degree of infiltration of tumor fibrous tissue and immune cells to obtain a comprehensive assessment of the overall TME.

### Evaluation of immunotherapy response and immune checkpoints

Therapy using immune checkpoint blockade is emerging as a promising strategy for cancer treatment [69]. In this study, we employed the Tumor Immune Dysfunction and Exclusion (TIDE) model to investigate the key mechanisms underlying tumor immune evasion and to forecast the therapeutic response of GM subtypes using expression profile data [70]. Moreover, we estimated the expression of programmed cell death 1 (PD1) and cytotoxic T-lymphocyte associated protein 4 (CTLA4) immune checkpoint molecules in different subtypes, which can regulate self-immune response by modulating T cell activity. This analysis allowed us to determine the sensitivity of different subtypes to immune checkpoint inhibitor therapy [71].

### Drug susceptibility analysis

For the evaluation of the therapeutic efficacy of the drugs in both subgroups, we utilized the “pRRophetic” R package to compute semi-inhibitory concentration (IC50) values corresponding to the drugs.

### Identification and analysis of prognosis-related GMRGs

We performed rigorous analyses using the TCGA-COAD dataset to determine GMRGs associated with prognosis, utilizing a univariate Cox regression analysis. To further assess the degree of relationship among these genes, we calculated Pearson correlation coefficients using the highly regarded “Hmisc” R package. The post-screening genes were then subjected to rigorous visualization and analysis using the powerful “maftool” package. In addition, we employed advanced R packages, such as “psych”, “GSVA”, and “ggcorrplot”, to comprehensively investigate the association between prognostic GMRGs and tumor-infiltrating immune cells. To elucidate the chromosomal localization of prognosis-associated GMRGs, we utilized the “RCircos” package. Finally, we conducted a thorough examination of the association between prognosis-related GMRGs and common immune checkpoints, providing a critical understanding of the potential impact of these genes on the immune response to cancer.

### Construction and evaluation of GM risk signature

On the basis of prognosis-associated genes by univariate Cox regression analysis, we performed multivariate Cox regression analysis on the TCGA-COAD dataset to construct a prediction model associated with GM and to obtain GMRBs. All CRC patients were stratified into high- and low-risk groups according to the median risk score calculated from TCGA-COAD data. The log-rank test was applied to investigate the difference in OS between the high- and low-risk groups, and the sensitivity and specificity of the features were evaluated by time-dependent receiver operating characteristic (ROC) curve analysis. The association between molecular consensus subtypes, consensus clustering subtypes, endpoint survival status, and risk models was also visualized by Sankey diagram.

### GMRBs survival analysis and immune correlation analysis

To investigate the potential prognostic implications of two GMRBs identified through multivariate Cox regression analysis, we correlated risk scores with the expression levels of these genes. Patients were divided into high- and low-expression cohorts according to median gene-expressed levels. Survival analysis was subsequently conducted for each group, and Kaplan-Meier (K-M) survival curves were constructed to visualize these results. To gain further insights into the underlying biological mechanisms driving the prognostic role of these genes, we examined their correlation with representative immune cells using the “ggcor” package.

### Expression analysis of GMRBs across diverse molecular subtypes, cancer types, and disease stages

We performed differential expression profiling of two GMRBs in various molecular subtype systems, encompassing consensus clustering, high-low risk clustering, and CRC consensus subtypes. This analysis was carried out employing the “ggpubr” and “ggsignif” software packages, with the goal of elucidating the association between GMRBs and the prognostic value within different subtypes. Furthermore, a comprehensive pan-cancer investigation of the GMRBs was conducted using the web-based tool “GEPIA” (http://gepia.cancer-pku.cn/). Additionally, we acquired differential expression data for the GMRBs in healthy individuals, patients diagnosed with V, and individuals with CRC, utilizing the GSE37283, TCGA-COAD, and GSE3629 datasets. Finally, within the TCGA-COAD dataset, we performed expression profiling of the GMRBs across distinct stages of CRC.

### Statistical analysis

Data analyses were carried out with R software v4.2.0. Pearson’s coefficient or Spearman’s coefficient was used to calculate the correlation between variables. Continuous variables fitted to a normal distribution between two groups were compared using *t*-tests. Analysis of variance was employed to compare more than two groups. Count data between groups were tested by χ2. All statistical calculations were evaluated by the following standards: ^*^P < 0.05; ^**^P < 0.01; ^***^P < 0.001.

## Supplementary Materials

Supplementary Figures

Supplementary Table 1

Supplementary Table 2
