# Supplementary material for: Identification of gut microbes-related molecular subtypes and their biomarkers in colorectal cancer
Source: Aging (Albany NY). 2024 Jan 29;16(3):2249–72. doi: 10.18632/aging.205480 (PMC10911361; doi:10.18632/aging.205480)
Supplement: Supplementary Figures [file aging-16-205480-s001.pdf]

SUPPLEMENTARY FIGURES

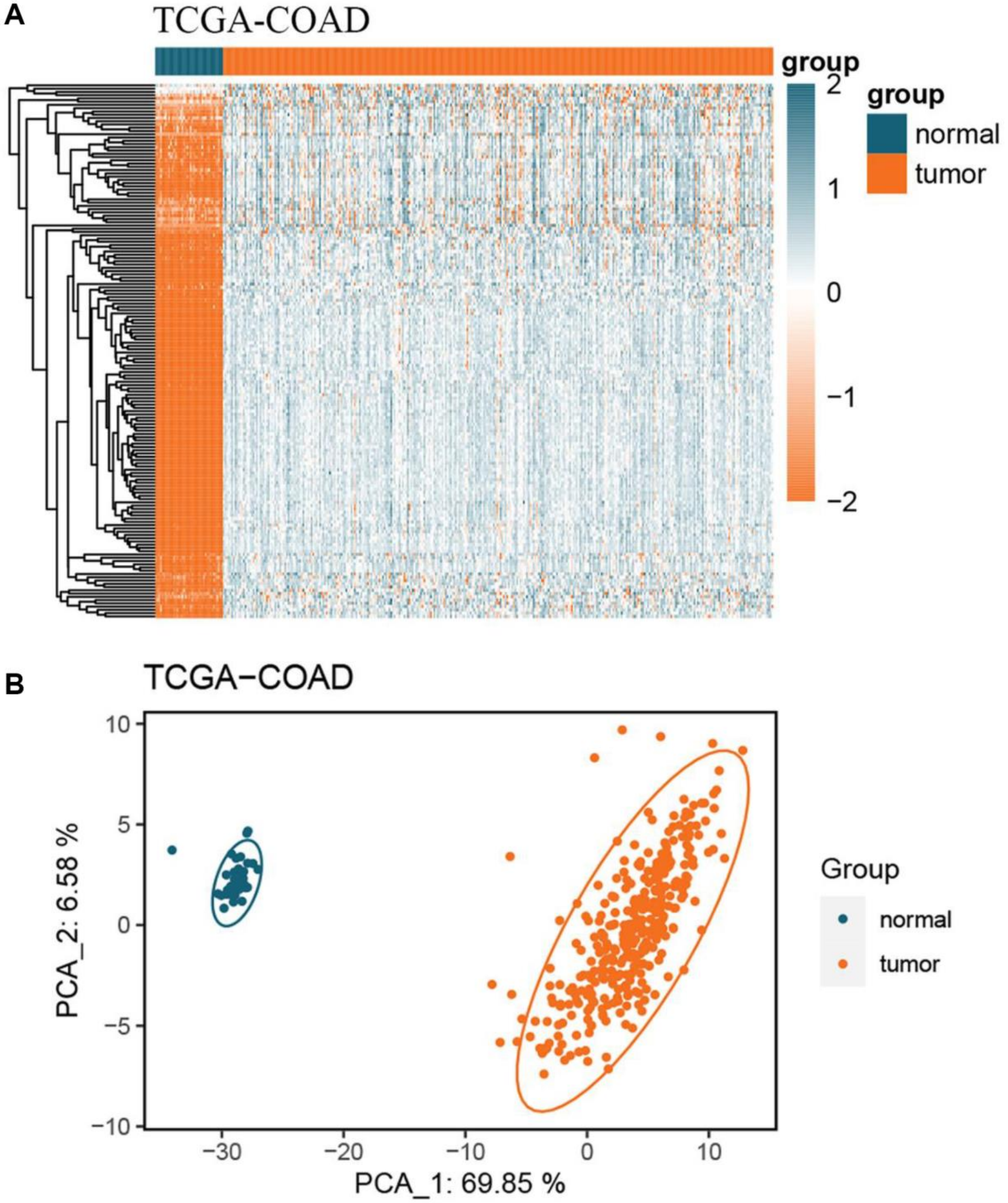

Supplementary Figure 1. Expression analysis of GMRGs in the TCGA-COAD cohort. (**A**) PCA plot of GMRGs. (**B**) Heatmap of GMRGs. Abbreviations: GMRGs: gut microbes-related genes; PCA: principal component analysis.

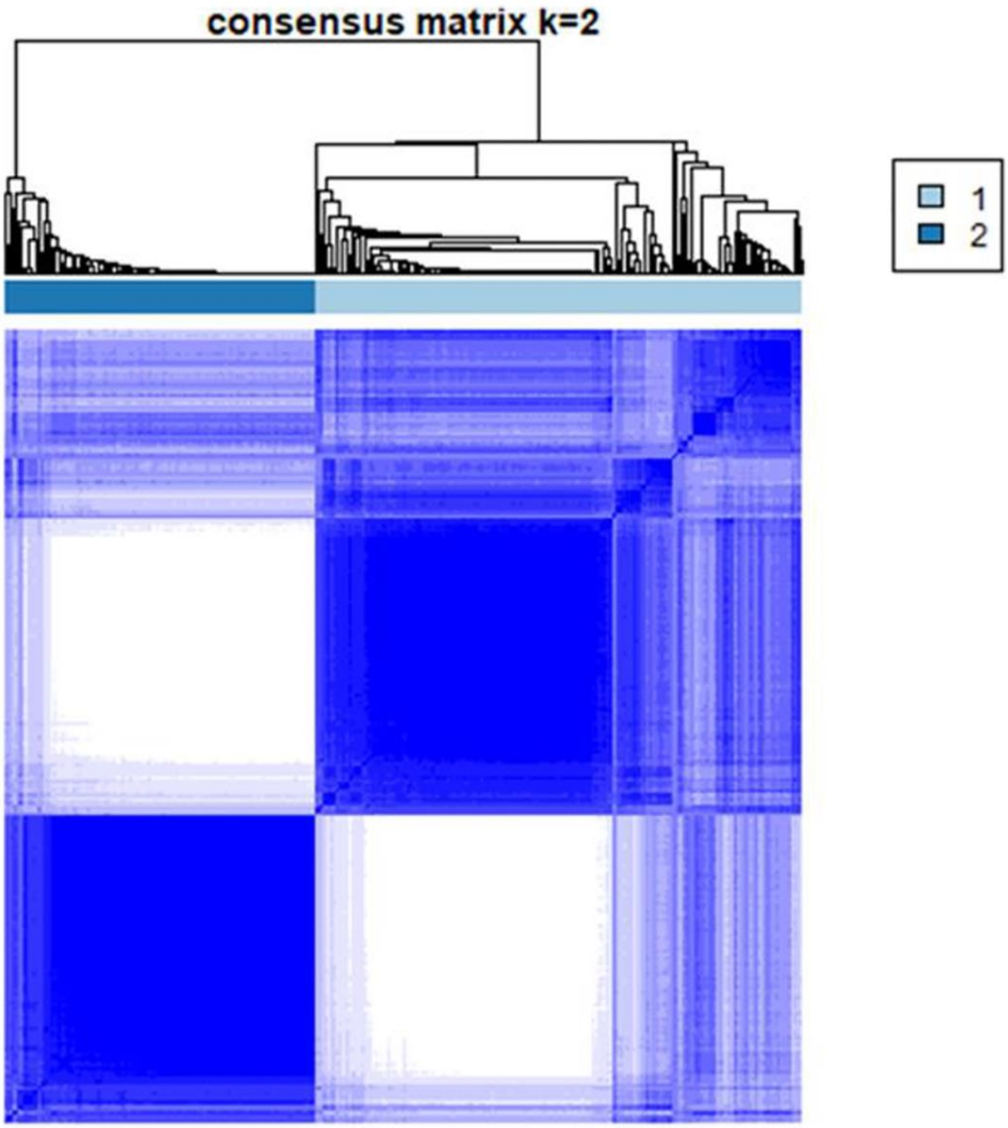

Supplementary Figure 2. Construction of GM molecular subtypes using TCGA-COAD samples. The two GM subtypes identified by the consensus clustering. Abbreviation: GM: gut microbes.

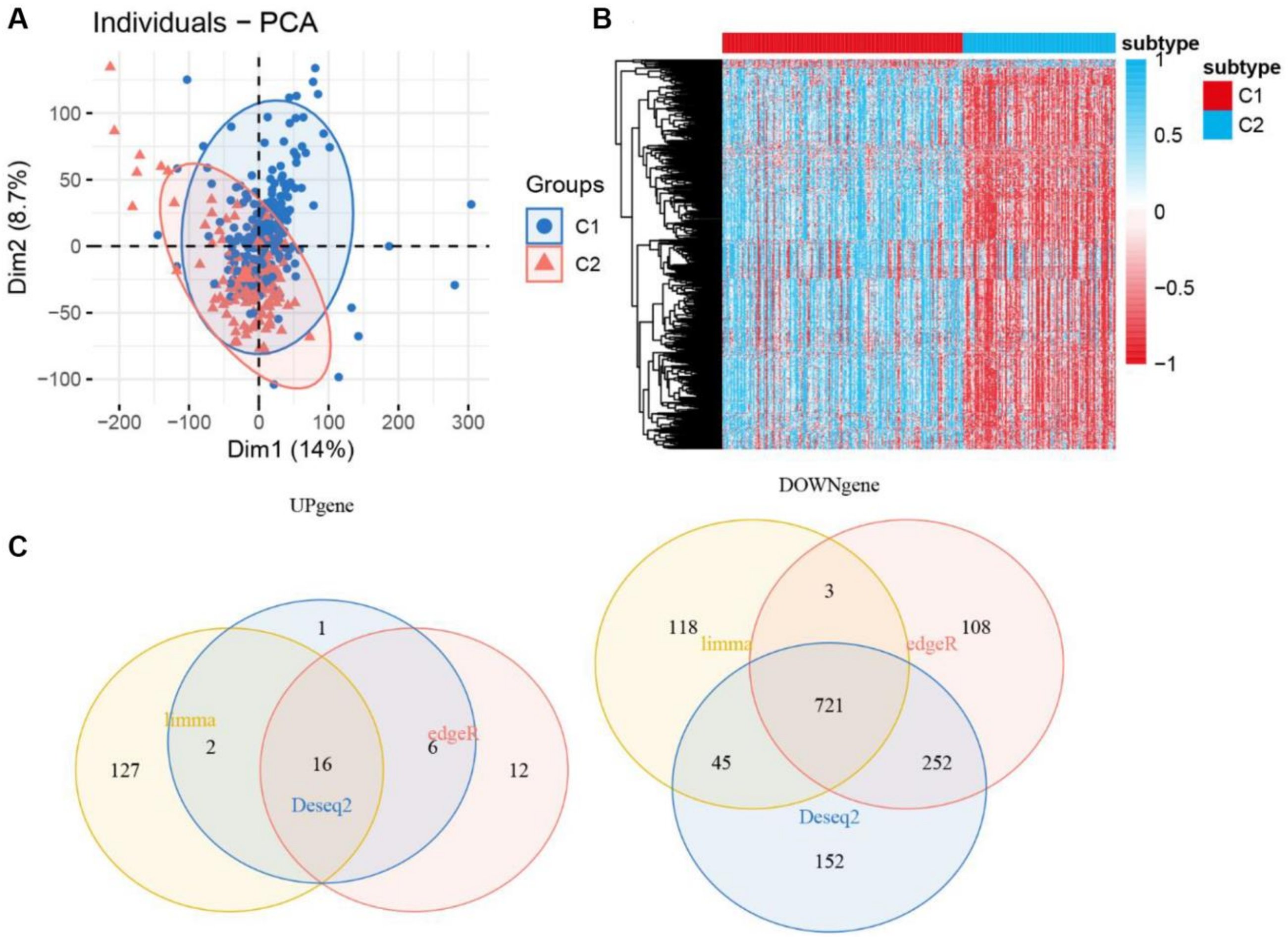

Supplementary Figure 3. DEGs between GM molecular subtypes. (**A**) PCA plot of DEGs. (**B**) Heatmap of DEGs. (**C**) Venn diagram of DEGs. Abbreviations: DEGs: differentially expressed genes; GM: gut microbes; PCA: principal component analysis.

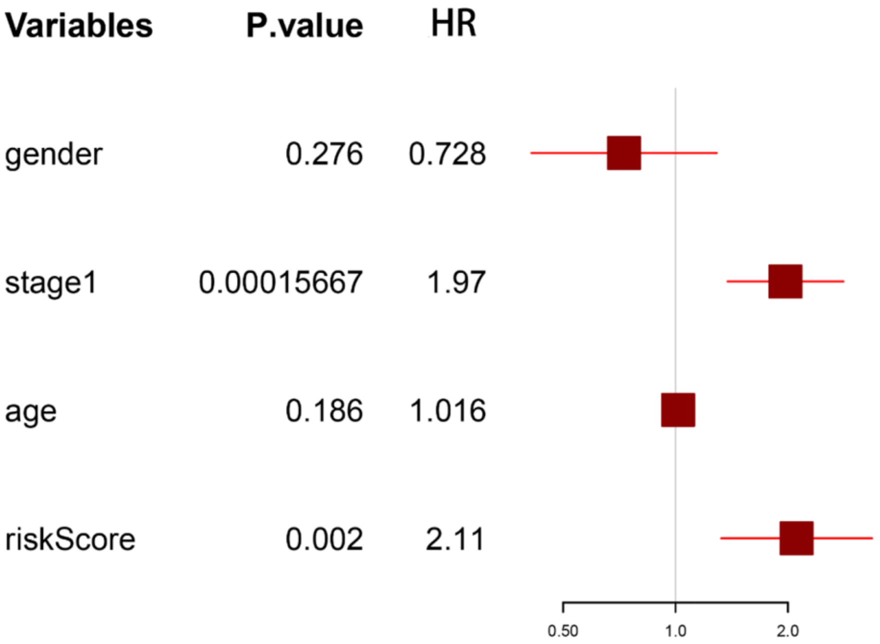

Supplementary Figure 4. Clinical value of risk signature. Forest plot of clinical characteristics and risk scores.

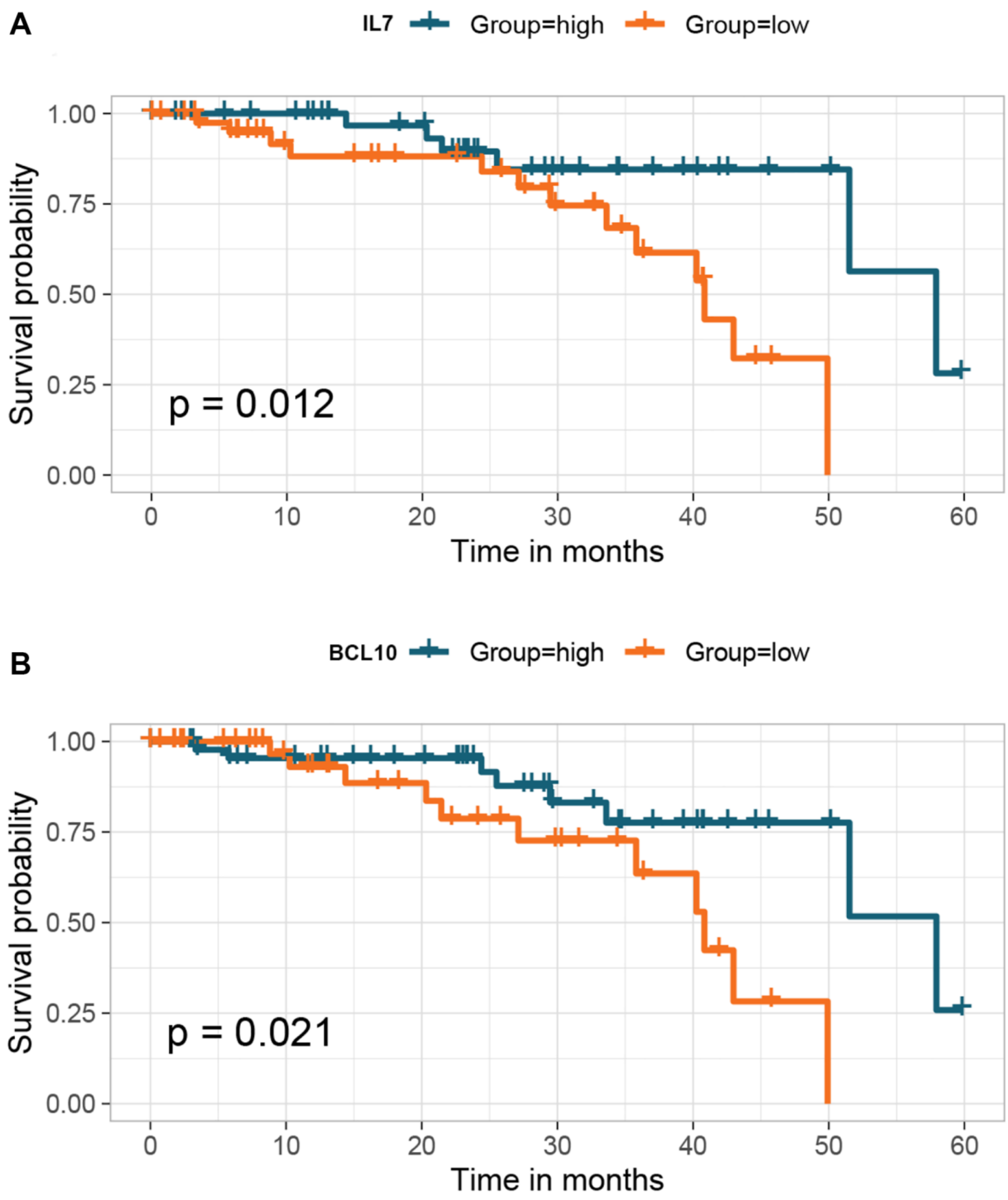

Supplementary Figure 5. K-M survival analysis based on GMRBs expression in the GSE87211 cohort. (**A**) K-M survival analysis between high and low expression groups of IL7. (**B**) K-M survival analysis between high and low expression groups of BCL10. Abbreviations: K-M: Kaplan-Meier; GMRBs: gut microbes-related biomarkers.
