# Supplementary material for: Identification of gut microbes-related molecular subtypes and their biomarkers in colorectal cancer
Source: Aging (Albany NY). 2024 Jan 29;16(3):2249–72. doi: 10.18632/aging.205480 (PMC10911361; doi:10.18632/aging.205480)
Supplement: Supplementary Table 1 [file aging-16-205480-s002.pdf]

SUPPLEMENTARY TABLE

Supplementary Table 1. Univariate Cox regression analysis identified 9 prognostic genes in the TCGA-COAD cohort (*P* < 0.05).

GENE	HR	*P*
MTOR	2.256	0.007
MMP9	2.012	0.025
NPC1L1	0.433	0.007
PKN2	1.853	0.038
PTGS2	2.064	0.027
SULT2B1	0.552	0.045
BCL10	2.248	0.007
STAT3	2.104	0.019
IL7	2.090	0.019
