# Supplementary material for: Identification of gut microbes-related molecular subtypes and their biomarkers in colorectal cancer
Source: Aging (Albany NY). 2024 Jan 29;16(3):2249–72. doi: 10.18632/aging.205480 (PMC10911361; doi:10.18632/aging.205480)
Supplement: Supplementary Table 2 [file aging-16-205480-s003.docx]

**Supplementary Table 2. 164 GMRGs obtained using a keyword search strategy in the PubMed database.**

| **NUMBER** | **GENE** | **PMID** |
| --- | --- | --- |
| 1 | CREBBP | PMID: 33653893  PMID: 25600494 |
| 2 | ADIPOR2 | PMID: 34019043 |
| 3 | NR1H4 | PMID: 32173762 |
| 4 | NPC1L1 | PMID: 36451858 |
| 5 | NR1H3 | PMID: 18088401  PMID: 27900259 |
| 6 | CASP8 | PMID: 30872395 |
| 7 | PKN2 | PMID: 35344714  PMID: 31451768 |
| 8 | TNFRSF1A | PMID: 36608535 |
| 9 | RHOA | PMID: 31736766 |
| 10 | ZONULIN | PMID: 28069576 |
| 11 | HNF4A | PMID: 37526424 |
| 12 | HLAC | PMID: 36416582  PMID: 36463279 |
| 13 | PTGS2 | PMID: 34700376  PMID: 35951774 |
| 14 | ICAM3 | PMID: 37289890 |
| 15 | ITCH | PMID: 36240781 |
| 16 | CEACAM1 | PMID: 33563644 |
| 17 | CXCL2 | PMID: 33495520 |
| 18 | SULT2B1 | PMID: 20579402 |
| 19 | CD209 | PMID: 19592647 |
| 20 | NLRC4 | PMID: 33452867 |
| 21 | NLRP1 | PMID: 30759441 |
| 22 | MSH2 | PMID: 32350866 |
| 23 | MAPK1 | PMID: 30930024 |
| 24 | MMP9 | PMID: 32017001 |
| 25 | TLR8 | PMID: 25896428 |
| 26 | XIAP | PMID: 34246792 |
| 27 | MAPK3 | PMID: 36841025 |
| 28 | UBE2I | PMID: 25097252 |
| 29 | MEFV | PMID: 29203393 |
| 30 | PYCARD | PMID: 31286804 |
| 31 | RIPK2 | PMID: 31286804 |
| 32 | IL7 | PMID: 32279195 |
| 33 | TGFB1 | PMID: 32805279 |
| 34 | CEACAM5 | PMID: 31585122 |
| 35 | CDK6 | PMID: 34742645 |
| 36 | AHR | PMID: 32901017 |
| 37 | CXCL12 | PMID: 29437871 |
| 38 | ICAM2 | PMID: 10898762 |
| 39 | CCL2 | PMID: 34999087 |
| 40 | NFKB1 | PMID: 32771947 |
| 41 | CCND1 | PMID: 32419125 |
| 42 | CBL | PMID: 33225894 |
| 43 | IL23A | PMID: 28823860 |
| 44 | VDR | PMID: 32194242 |
| 45 | IFNG | PMID: 34358434 |
| 46 | PTPN6 | PMID: 32403971 |
| 47 | CLEC4A | PMID: 27694959 |
| 48 | MAPK14 | PMID: 27995407 |
| 49 | IL17A | PMID: 33859636 |
| 50 | CCND3 | PMID: 10716680 |
| 51 | MSH3 | PMID: 22457395 |
| 52 | NR3C1 | PMID: 32258258 |
| 53 | CD4 | PMID: 30939976 |
| 54 | CCL20 | PMID: 34632963 |
| 55 | IL18R1 | PMID: 11788557 |
| 56 | HDAC1 | PMID: 29317660 |
| 57 | RPS6KA1 | PMID: 34099716 |
| 58 | CNR1 | PMID: 36902274 |
| 59 | PIK3CA | PMID: 34108031 |
| 60 | CXCR4 | PMID: 37101548 |
| 61 | PKN1 | PMID: 32764209 |
| 62 | NR4A1 | PMID: 18088401  PMID: 32165624 |
| 63 | CDK2 | PMID: 20979106  PMID: 33231228  PMID: 35151160 |
| 64 | CDKN1A | PMID: 35580023 |
| 65 | P2RX7 | PMID: 28297661 |
| 66 | IL1B | PMID: 33557671  PMID: 34612661 |
| 67 | FFAR2 | PMID: 20979106  PMID: 31628054 |
| 68 | HIF1A | PMID: 22157238 |
| 69 | SIRT1 | PMID: 36014845 |
| 70 | KDR | PMID: 33765061 |
| 71 | RIPK3 | PMID: 33769187  PMID: 29042502 |
| 72 | NR1H2 | PMID: 27900259 |
| 73 | FMO5 | PMID: 33413501 |
| 74 | PPARG | PMID: 37616368 |
| 75 | KRAS | PMID: 33145304 |
| 76 | CCNB1 | PMID: 25857357 |
| 77 | HRH4 | PMID: 35895832 |
| 78 | HNF1A | PMID: 27807544 |
| 79 | MAP3K7 | PMID: 33894128 |
| 80 | GPR55 | PMID: 31202201 |
| 81 | IL6 | PMID: 35788907 |
| 82 | IL10 | PMID: 36398889  PMID: 15845454 |
| 83 | REG3G | PMID: 29411774  PMID: 34135557 |
| 84 | TLR4 | PMID: 34784980  PMID: 34589510 |
| 85 | RIPK1 | PMID: 33769187 |
| 86 | TLR2 | PMID: 36398889  PMID: 10880445 |
| 87 | SOCS3 | PMID: 31221818  PMID: 23892476 |
| 88 | CASP1 | PMID: 24919149  PMID: 29437871 |
| 89 | CXCL9 | PMID: 29437871  PMID: 34964882 |
| 90 | CASP6 | PMID: 37603159  PMID: 34740613 |
| 91 | LEF1 | PMID: 31734354  PMID: 28670499  PMID: 37160722 |
| 92 | ESR2 | PMID: 20519411  PMID: 15883431 |
| 93 | SOD1 | PMID: 32555166  PMID: 36465648 |
| 94 | AKT1 | PMID: 33615993  PMID: 37100057 |
| 95 | BCL10 | PMID: 17540779 |
| 96 | NR1I3 | PMID: 18088401 |
| 97 | RORC | PMID: 18088401 |
| 98 | NR1I2 | PMID: 31998605  PMID: 35073747  PMID: 25065623 |
| 99 | EGFR | PMID: 15480783  PMID: 22704618 |
| 100 | NR6A1 | PMID: 18088401 |
| 101 | LCN2 | PMID: 32161843  PMID: 27078067  PMID: 31992345 |
| 102 | ADM | PMID: 10225288  PMID: 27965594  PMID: 27345325 |
| 103 | DRD2 | PMID: 36993486  PMID: 24047867 |
| 104 | IL18 | PMID: 33557671 |
| 105 | CXCL13 | PMID: 20643338  PMID: 37156441 |
| 106 | BRAF | PMID: 31555583  PMID: 26811607  PMID: 33382354 |
| 107 | FCER1G | PMID: 18227164  PMID: 26523352 |
| 108 | RUNX1 | PMID: 37834058  PMID: 32911536 |
| 109 | ADIPOR1 | PMID: 24520358 |
| 110 | ITGB2 | PMID: 36699448  PMID: 15837813  PMID: 11726969 |
| 111 | IL6R | PMID: 35982604  PMID: 33557671 |
| 112 | NAPEPLD | PMID: 33986673 |
| 113 | IL23R | PMID: 36384110 |
| 114 | NLRP3 | PMID: 32529941  PMID: 35844801 |
| 115 | AIM2 | PMID: 22430785  PMID: 30209070 |
| 116 | DCLK3 | PMID: 35619716 |
| 117 | CXCL3 | PMID: 22250091 |
| 118 | CXCL5 | PMID: 29934568 |
| 119 | CXCL1 | PMID: 29398651 |
| 120 | F2RL1 | PMID: 37131291  PMID: 31495063 |
| 121 | HTR4 | PMID: 36326009 |
| 122 | CASP3 | PMID: 3376918 |
| 123 | SYK | PMID: 32152943  PMID: 15845454 |
| 124 | CASP7 | PMID: 21296981  PMID: 21831793 |
| 125 | CLEC4E | PMID: 32540999 |
| 126 | CTNNB1 | PMID: 32795675  PMID: 30543778 |
| 127 | FADD | PMID: 33769187  PMID: 10880445 |
| 128 | CX3CR1 | PMID: 30503509  PMID: 29326275 |
| 129 | STAT3 | PMID: 36398889 |
| 130 | CXCL10 | PMID: 35259052  PMID: 26776522 |
| 131 | CXCL8 | PMID: 34700376  PMID: 23137964 |
| 132 | ITGAM | PMID: 12719479  PMID: 32152943 |
| 133 | CDK1 | PMID: 20979106  PMID: 33231228 |
| 134 | NMUR1 | PMID: 35139379  PMID: 37383227  PMID: 37383227 |
| 135 | BCL2 | PMID: 19276343 |
| 136 | MALT1 | PMID: 21334257  PMID: 21267996 |
| 137 | CLEC7A | PMID: 22674328  PMID: 19223162 |
| 138 | MYD88 | PMID: 34784980  PMID: 34589510 |
| 139 | OLR1 | PMID: 23085345  PMID: 11290792 |
| 140 | TFF3 | PMID: 31213556  PMID: 32663105 |
| 141 | NLRP6 | PMID: 35803930  PMID: 32855216 |
| 142 | IRF4 | PMID: 37161603  PMID: 34867956 |
| 143 | IL17RA | PMID: 34185157 |
| 144 | PTPN11 | PMID: 11743164 |
| 145 | GPBAR1 | PMID: 34329568  PMID: 28602676 |
| 146 | CXCR2 | PMID: 33323397 |
| 147 | F2R | PMID: 22407318  PMID: 37564290 |
| 148 | ADGRB1 | PMID: 26838550  PMID: 21245295 |
| 149 | ANXA2 | PMID: 23931152 |
| 150 | HCAR2 | PMID: 19276343 |
| 151 | GPR132 | PMID: 29576437  PMID: 26283367 |
| 152 | FFAR4 | PMID: 37408362 |
| 153 | RXRA | PMID: 16556443  PMID: 33022571 |
| 154 | PPARA | PMID: 33477821  PMID: 35105664 |
| 155 | TLR5 | PMID: 35668113 |
| 156 | CARD9 | PMID: 33548172  PMID: 30231985 |
| 157 | CNR2 | PMID: 33186639 |
| 158 | HDAC2 | PMID: 12840228  PMID: 29317660 |
| 159 | TRPV1 | PMID: 36240781  PMID: 33779497 |
| 160 | HCAR1 | PMID: 30543778 |
| 161 | MUC2 | PMID: 27863247  PMID: 36870625 |
| 162 | MTOR | PMID: 32989095 |
| 163 | AGER | PMID: 35580023;  PMID: 31711923 |
| 164 | TNF | PMID: 33268961 |

Abbreviations: GMRGs gut microbes-related genes.
